# Chemical Composition Analysis and Assessment of Antioxidant and Anti-Inflammatory Activities of Crude Extract of *Flueggea leucopyrus* on Carrageenan-Induced Paw Edema in Wistar Albino Rats

**DOI:** 10.3390/antiox13080976

**Published:** 2024-08-12

**Authors:** Vijayakumar Mayakrishnan, Anand Thirupathi, Kavitha Ramamoorthy, Kaliappan Annadurai, Radha Prakasam, Yaodong Gu, Choon Young Kim, Mahadevi Ramasamy, Habeebmon Karimpanchola, Priya Kannappan, Natesan Vijayakumar, Bhuvaneshwari Venkatesan Kumari, Anand Singaravelu

**Affiliations:** 1 Research Academy of Medicine Combining Sports, Ningbo No 2 Hospital, Ningbo 315010, China; marulbiochem@rediffmail.com (V.M.); anand@nbu.edu.cn (A.T.); guyaodong@nbu.edu.cn (Y.G.); 2 Faculty of Sports Science, Ningbo University, Ningbo 315211, China; 3 Research Institute of Human Ecology, Yeungnam University, Gyeongsan 38541, Gyeongbuk, Republic of Korea; 4 Department of Biotechnology, Periyar University, Salem 636 011, Tamil Nadu, India; mahadevi16ramasamy@gmail.com; 5 Department of Biotechnology, Periyar University Centre for Post Graduate and Research Studies, Dharmapuri 635 205, Tamil Nadu, India; kaliyappan5g@gmail.com; 6 Siddha Medicinal Plants Garden, (Central Council for Research in Siddha, Ministry of Ayush, Government of India), Mettur Dam, Salem 636 401, Tamil Nadu, India; radhasudar@rediffmail.com (R.P.); kchabeebmngd@gmail.com (H.K.); 7 Department of Food and Nutrition, Yeungnam University, Gyeongsan 38541, Gyeongbuk, Republic of Korea; cykim@yu.ac.kr; 8 Department of Biochemistry, PSG College of Arts and Science (Autonomous), Affiliated to Bharathiar University, Coimbatore 641014, Tamil Nadu, India; priya@psgcas.ac.in; 9 Department of Biochemistry and Biotechnology, Faculty of Science, Annamalai University, Annamalainagar 608002, Tamil Nadu, India; nv12507@annamalaiuniversity.ac.in (N.V.); bhuvibhuvi444@gmail.com (B.V.K.); 10 Department of Chemistry, Saveetha Engineering College (Autonomous), Saveetha Nagar, Thandalam, Chennai 602105, Tamil Nadu, India; anand.singaravelu@gmail.com

**Keywords:** *Flueggea leucopyrus*, phytochemical, antioxidant, GC–MS, anti-inflammatory

## Abstract

A member of the Phyllanthaceae family, *Flueggea leucopyrus* is a well-known plant in the tribal areas of Sri Lanka, India’s Shaurastra region, Australia, and Malaysia. This study provides information about *Flueggea leucopyrus*, a plant with a wide range of therapeutic uses in India. Different extracts from the leaves and roots of *Flueggea leucopyrus* were evaluated for their physical and chemical properties, preliminary phytochemical parameters, and pharmacological activities in the current study, followed by their fourier transform infrared spectroscopy (FTIR), gas chromatography-mass spectrometry (GC–MS), antioxidant, and anti-inflammatory properties. The aqueous extract of *Flueggea leucopyrus* leaves and roots have more different phytochemical elements than other solvent extracts, according to physico-chemical tests and phytochemical screening. As a result, the FT-IR, GC–MS, antioxidant, and anti-inflammatory activities of an aqueous extract were tested. Studies on hind paw edemas caused by carrageenan in albino rats examined the mean increase in paw volume and the percentage inhibition in paw volume at various time points following the injection of carrageenan (1% *w*/*v*). In comparison to the norm, these inhibitions were statistically significant (*p* < 0.001). The aqueous extract of *Flueggea leucopyrus* leaves and roots have both antioxidative and anti-inflammatory activities, indicating that it has the potential to be used in the formulation of antioxidant and anti-inflammatory medications in the future.

## 1. Introduction

A pathologic reaction to damage, infection, or destruction, inflammation is marked by heat, redness, discomfort, swelling, and abnormal functioning. Inflammation is a common protective response to tissue injury caused by physical trauma, toxic chemicals, or microbial infections. The body reacts in order to neutralize or eliminate the invaders, rid itself of the irritants, and prepare the ground for tissue restoration. According to Estina et al., it is brought on by the release of chemical mediators from damaged tissue and migratory cells [1]. Unwanted pathologic illnesses such as rheumatoid arthritis, neurodegenerative diseases, cancer, asthma, and inflammatory bowel disease are brought on by excessive and prolonged inflammation, which is one of the causes of inflammation [2]. 

Non-steroidal anti-inflammatory medicines (NSAIDs) and corticosteroids are two medications frequently used to treat inflammatory disorders. Long-term use of NSAIDs, such as aspirin and coxibs, raises the risk of gastrointestinal and cardiovascular disorders [3]. This raises the question of whether any natural substances that could imitate the effects of synthetic NSAIDs while having fewer negative effects. By scavenging free radicals, antioxidants provide protective benefits against oxidative stress [4]. Synthetic antioxidants for regulating unwanted redox processes are expensive and frequently plagued by unavailability and negative effects. Due to these limitations, natural antioxidants have attracted interest throughout history because they are more economical, do not have the undesirable side effects of their synthetic counterparts, and are found in many plants [5]. 

Natural component formulations have been presented as a viable solution to some of these issues. Natural compounds are often the first-line defence in treating both acute and chronic health conditions due to their ease of use, efficacy, and claimed low adverse effects [6]. As a result of these characteristics, diverse medicinal plants that have been discovered to have diverse phytochemical constituents with analgesic and anti-inflammatory actions are commonly utilized in the treatment of pain and chronic illnesses [7]. In plants, a variety of phytochemicals work together in harmony to produce the pharmacological effects that are observed. The species *Flueggea leucopyrus* (*F. leucopyrus*) Willd (Katupila) is a member of the Phyllanthaceae family. In Sri Lankan traditional medicine, *F. leucopyrus* leaves have been used to cure cancer, boils, external ulcers, and wounds [8]. Traditional medicinal preparations of the plant have been used to treat kidney stones, mental disorders, gonorrhea, digestive difficulties, coughs, hay fever, as disinfectants, and coughing fits [9]. The main method for obtaining these bioactive chemicals from biomass material is an extraction procedure [10]. Only a few studies have looked into how different solvents affect the amount of bioactive chemicals that can be extracted from *F. leucopyrus* and their pharmacological effects. The current study examined the effects of physico-chemical characteristics, extractive values of various solvents (ethanol, methanol, and aqueous), and qualitative and quantitative phytochemical analysis before examining the antioxidant and anti-inflammatory properties of *F. leucopyrus*.

## 2. Materials and Methods

### 2.1. Collection and Authentication of Plant Material

We collected mature and healthy *F. leucopyrus* leaves and roots from Mettur Dam in the Salem district of Tamil Nadu, India, to be used for this study. Research Officer (Botany) for the Central Council for Research in Siddha, Government of India, Mettur Dam, Salem, Tamil Nadu, India, authenticated the collection of plants. During two weeks, leaf and root samples were washed with distilled water and dried at a temperature between 27 and 37 °C in the complete absence of sunlight. In order to further analyze the leaf and root samples, they were dried and ground to produce a fine powder. The powder was packed into airtight bottles and kept at room temperature.

### 2.2. Experimental Animals

We received normal healthy adult Wistar albino rats from Arulmigu Kalasalingam College of Pharmacy that weighed 160–180 grammes and were roughly the same age. The Institutional Animal Ethics Committee (IAEC), a committee for the purpose of animal experimentation (approval number: AKCP/IAEC/23/21-22) that is certified by the Committee for the Purpose of Control and Supervision of Experiments on Animals (CPCSEA), approved the entire process at the Arulmigu Kalasalingam College of Pharmacy in Krishnankoil, Tamil Nadu, India. Animals were housed in a well-ventilated animal house at 24–28 °C temperature with a 12 h light–12 h dark cycle and kept in clean and dry polycarbonate cages. Animals were fed a regular pellet diet (bought from Sai Durga Feeds in Bangalore, India) and had access to free water throughout the trial.

### 2.3. Preparation of Plant Extract

Several weeks were spent in the shade drying the *F. leucopyrus* leaf and root samples that had been freshly collected. The leaves and roots were cleaned and sliced into small pieces after being washed in distilled water. The leaves were separated after they had been shading dried and roughly pulverized. The powdered components were then stored in airtight containers until we were ready to use them. We processed 500 g of dried coarse powdered materials with 1250 mL of methanol, ethanol, and water for 24 h in a Soxhlet extractor. We filtered each extract individually using Whatmann No. 41 filter paper and concentrated the extracts under vacuum in a rotary evaporator at 60 °C. We dried the extracts in an oven at a temperature of 40–50 °C for 8 h to remove the remaining solvent, and then we used the extracts for qualitative identification of different phytochemical constituents and pharmacological studies according to standard procedures [11].

### 2.4. Determination of Physicochemical Properties

The following method was used [11] to conduct various physico-chemical investigations on coarsely powdered sun-dried crude drugs of *F. leucopyrus* leaves and roots.

### 2.5. Fluorescence Analysis in Dried Powder 

Different ingredients of powdered plant material will fluoresce differently under ultraviolet radiation. Every characteristic of the fluorescence of each sample was observed under ordinary light and then under UV light of both long and short wavelengths. *F. leucopyrus* leaf and root powders were analyzed for fluorescence using a variety of chemical treatments. Furthermore, even if the substances themselves are not luminous, they can frequently be transformed into fluorescent derivatives or degradation products by using various chemicals. The fluorescence characteristics of the samples were observed both under ordinary lighting and under long and short wavelength UV light. They were all mixed with acids viz. powder + 1N NaOH, powder + 1N HCl, powder + Conc. HCl, powder + Conc. H_2_SO_4_, powder + 50% HNO_3_, powder + 40% NaOH, powder + 10% lead acetate, powder + acetic acid, powder + FeCl_3_, powder + petroleum ether, powder + acetone, powder + chloroform, powder + ethanol, powder + methanol, and powder + ammonia [12].

### 2.6. Phytochemical Characterization 

*F. leucopyrus* leaf and root extracts were tested in methanol, ethanol, and aqueous extracts for a variety of phytoconstituents.

The qualitative phytochemicals were tested for alkaloids (Mayer’s reagent), flavonoids (alkaline reagent), phenols (ammonium hydroxide test), fixed oil (spot test), quinines (sulfuric acid test), saponins (foam test), steroids (Salkowski’s test), carbohydrates (Benedict’s test), tannins (ferric chloride test), starch, coumarin, resins, proteins, cardiac glycosides (Keller–Killani’s test), anthocyanin, and terpenoids (Libermann burchand test) using standard protocol [13]. A quantitative analysis of phytochemicals and nutrients found in the aqueous extracts of the analyzed plant was conducted using the following tests: alkaloids were quantified using an ammonium hydroxide precipitation test, flavonoids were analyzed using a spectrophotometer against rutin equivalent, total carbohydrates were estimated using Anthrone’s method, and total proteins was estimated by Lowry’s method. Total phenolics and standard gallic acid equivalents were calculated at 765 nm under spectrophotometer [13].

### 2.7. FT-IR Spectrum Analysis

An FT-IR analysis was performed using a Perkin Elmer Spectrophotometer instrument to detect the distinctive peaks in addition to their functional groups. In order to analyze the FT-IR spectrum, the extracts were centrifuged at 3000 rpm for about 10 min, followed by filtration on Whatmann No. 1 filter paper by means of a high-pressure vacuum pump. The extracts were scanned with a Perkin Elmer Spectrophotometer on a wavelength range of 300–1100 nm, and their sharp peaks were found after being diluted to 1:10. We captured the peak FT-IR values and repeated each analysis twice for spectrum confirmation [13].

### 2.8. GC–MS Analysis

Gas chromatography–mass spectrometry (GC–MS) is essential for analyzing unknown plant components. The chemical compositions of *F. leucopyrus* leaf and root extracts were investigated using GC–MS. To undertake GC–MS analysis on such samples, a Perkin Elmer GC clarus 500 system with an AOC-20i autosampler and gas chromatograph linked up to a mass spectrometer was used (GC–MS). In our experiment, we utilized a Fusion Silica Elite-1 column (30 m × 0.25 mm ID × 15 mm DL dimension), operating in electron impact mode at 70 eV with helium gas (99.99%) as the carrier gas and an injection volume of 2 L (split ratio 10:1). The injector was set to 250 °C, the ion source to 280 °C, and the oven to 110 °C (isothermal for 2 min), with a 10 °C min^−1^ increase set for this temperature to 200 °C min^−1^, then 5 °C min^−1^ to 280 °C min^−1^, concluding with a 9 min isothermal at 280 °C. GC ran at 70 eV with a scanning interval of 0.5 s, with fragment sizes ranging from 45 to 450 Da. The relative amount of each component was calculated by comparing its peak area to the total area of the spectrum. 

As a software tool to handle mass spectra and chromatograms, Turbo Massver 5 was used and the interpretation of the mass spectrums of the GC–MS was completed based on the National Institute of Standards and Technology (NIST) database, which contains more than 62,000 patterns. When the unknown component’s spectra were compared to those in the NIST library, molecular weight and structure were determined [14].

### 2.9. In-Vitro Antioxidant Potential

We evaluated the antioxidant activity of *F. leucopyrus* in vitro was determined through the following methodology [15].

### 2.10. Acute Anti-Inflammatory Activity

In the case of paw edema induced by carrageenan, a classic test for acute anti-inflammatory agents has been developed, and its injection in rats causes biphasic inflammation that can be separated into early and late phases. During the first hour, inflammatory mediators such serotonin, histamine, and bradykinins are released. A late phase of carrageenan-induced inflammation develops following inflammation, when early-phase mediators engage pathways that promote neutrophil infiltration and additional prostaglandin generation. In addition to neutrophil-derived free radicals, nitric oxide and pro-inflammatory cytokines such as interleukin-1b (IL-1b) and tumor necrosis factor-a (TNF-a) are promoted. 

Carrageenan was used to induce rat foot edema for the evaluation of acute anti-inflammatory activity [16]. In eight groups of six animals each, the rats were divided after fasting for 12 h, and each one was marked with a unique identification number. In the control group, Group I, only carrageenan was administered, whereas in Group II, the standard drug compound indomethacin at a dosage rate of 3 mg kg^−1^ of body weight was administered orally. In Groups III, IV, and V, the aqueous leaf extract of *F. leucopyrus* was administered at dosages of 100, 250, and 500 mg kg^−1^ orally; and in Group VI, VII, and VIII, the aqueous root extract of *F. leucopyrus* was administered orally at dosages of 100, 250, and 500 mg kg^−1^ orally. Half an hour before administering the carrageenan, the animals were treated with the extract. After receiving subplantar injections of 1% carrageenan in normal saline, both the experimental and control rats developed acute inflammation in the right paw. The paw was marked and immersed in mercury up to the level of lateral malleous and measured by mercury volume displacement methods. Following the injection of carrageenan into each group, the paw volume was measured after 15 min, 30 min, 1 h, 2 h, and 4 h. The variance in measurements was used to determine edema and the percentage of anti-inflammatory action was computed.

% of inhibition rate = Ve − Vt/Ve × 100

where Ve is the edema value of the control group and V is the edema value of the treated groups.

### 2.11. Statistical Analysis

All experiments were performed in triplicate (*n* = 3) and the results were expressed as mean ± SEM. Statistical analyses were carried out with SPSS (package version 17.0) using one-way analysis of variance (ANOVA). Significant differences were calculated according to the Tukey’s test. A correlation analysis of the results was performed in SPSS and significant differences were considered statistically significant at the level of *p* < 0.05.

## 3. Results

For the treatment of a wide range of illnesses, the genus *F. leucopyrus* has been mentioned in several traditional healthcare systems. The leaves and roots of *F. lecopyrus* have been examined for their physico-chemical, antioxidant, and anti-inflammatory capabilities in the current study.

### 3.1. Physico-Chemical Properties in Dried Leaf Powder of Leaf and Root of F. leucopyrus

The physico-chemical characteristics of the leaf and root powders of *F. leucopyrus* is depicted on Table 1. Moisture content was found to be low in both leaf and root samples. The amount of total ash (4.16 ± 0.07%), acid-insoluble ash (3.47 ± 0.08%), and sulphate ash (5.75 ± 0.14%) were higher in the root sample compared to the leaf sample. The percentage of extractive values of crude drugs was higher in aqueous and methanol in comparison to other solvents. The crude fiber content of the leaf and root samples of *F. leucopyrus* were found to be 10.23 ± 0.14% and 11.63 ± 0.11%, respectively.

### 3.2. Fluorescence Analysis

This study also helps to characterize crude drugs by analyzing the behavior of the powdered plant materials of *F. leucopyrus* leaves and roots treated with different chemical reagents observed in daylight as well as under UV light at 254 nm and 365 nm. These data are summarized in Table 2.

### 3.3. Preliminary Phytochemical Characterization of F. leucopyrus

As shown in Table S1, the distribution of different phytochemical compositions was measured qualitatively in methanol, aqueous, and ethanol extracts of the leaves and roots of *F. leucopyrus*. It showed positive results for alkaloids, flavonoids, phenols, fixed oil, quinines, saponins, steroids, carbohydrates, tannins, starch, coumarin, resins, proteins, cardiac glycosides, and anthocyanin in all extracts using the standard protocol [13]. The aqueous extracts of leaves and roots showed positive results for alkaloids, flavonoids, phytosterol, saponins, steroids, carbohydrates, tannins, terpenoids, coumarin, protein, and anthocyanin, and the ethanol extracts of leaves and roots showed positive results for flavonoids, glycosides, phenols, saponins, and starch. These findings proved that the maximum phytoconstituents are present in the aqueous extract of the leaves and roots of *F. leucopyrus*.

### 3.4. Quantification of Phytochemicals and Nutrients Present in Aqueous Extracts of F. leucopyrus

Table 3 showd the quantitative analysis of phytochemicals and nutrients found in the aqueous extracts of the analyzed plant [13]. The results provided an accurate quantitative measurement of the *F. leucopyrus* leaf and root aqueous extract. Alkaloids (18.53 ± 0.12 mg gm^−1^), carbohydrates (71.34 ± 0.09 mg gm^−1^), and total phenolic content (78.90 ± 0.09 mg gm^−1^) were highly present in the root extract compared to the leaf extract. The flavonoid (45.32 ± 0.16 mg gm^−1^) and protein (32.67 ± 0.15 mg gm^−1^) contents were rich in the leaf extract compared to the root extract. The existence of these phytochemical substances in the selected medical plants has a wide range of uses and may undoubtedly be employed for a variety of purposes. The findings of this study imply that the detected phytochemical compounds are the bioactive ingredients, and that this plant, *F. leucopyrus*, is proving to be an increasingly useful reservoir of bioactive compounds with significant therapeutic value. Quantitative study of these essential components is critical for determining medication quality [13].

### 3.5. FT-IR Spectroscopy Analysis

**Figure 1:**
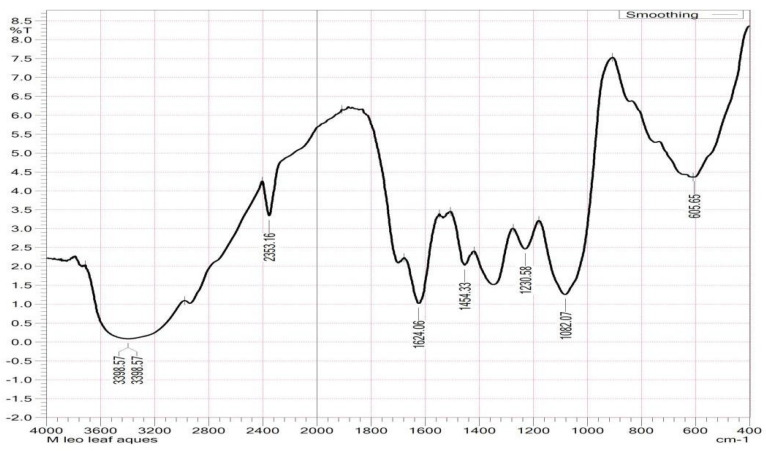
FT-IR chromatogram of the *F. leucopyrus* leaf extract.

**Figure 2:**
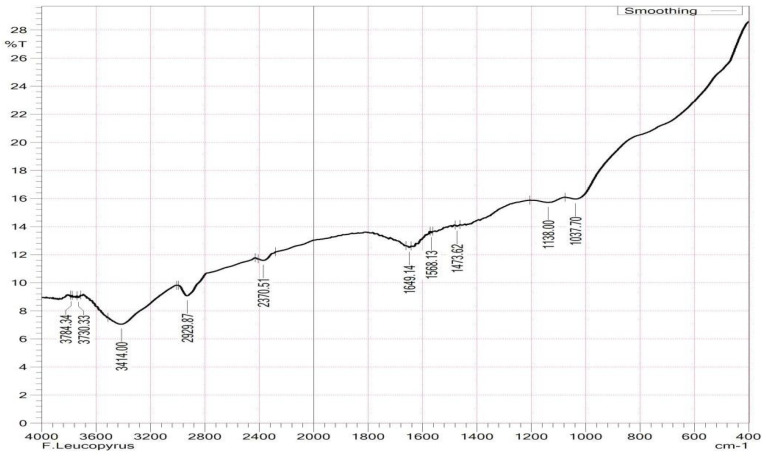
FT-IR chromatogram of the *F. leucopyrus* root extract.

FT-IR is effective for detecting biomolecules from plant extracts, and we employed it for analyzing the crude aqueous leaf and root extracts of *F. leucopyrus*. Based on peak ratios, functional groups were separated, and Table 4 and Figure 1 and Figure 2 show the FT-IR spectrogram profile of the aqueous extracts of *F. leucopyrus* leaves and roots, along with the characteristic absorption peaks. The FT-IR spectrum exhibited strong absorption bands at IR (KBr)ʋ cm^−1 bt^. From Table 4, alcohols and phenols (O-H stretching, H-bonded) showed peaks at 3414.00 cm^−1^, 3784.34 cm^−1^, and 3730.33 cm^−1^ in the root extract, whereas it peaked at 3398.57 cm^−1^ in the leaf extract. Primary amines (N-H bend) showed peaks at 1624.06 and 1649.14 cm^−1^ in the leaf and root extracts. Aldehydes (H-C=O: C-H stretching) showed peaks at 2353.16 cm^−1^ and 2370.51 cm^−1^ in leaf and root extracts. Aliphatic amines (C-N stretching) showed peaks at 1230.58 cm^−1^ and 1082.07 cm^−1^ in the leaf extract and 1138.00 cm^−1^ and 1037.70 cm^−1^ in the root extract, respectively. Alkanes (C-H bend) exhibited a peak at 1454.33 cm^−1^ in the leaf extract, whereas alkanes exhibited a peak at 2929.87 cm^−1^ only in the root extract. Aromatics (C-C stretching) showed peaks at 1568.13 cm^−1^ and 1473.62 cm^−1^ in the root extract, whereas alkyl halides (C-Br stretching) detected peaks at 605.65 cm^−1^ in the leaf extract.

### 3.6. GC–MS Analysis

**Figure 3:**
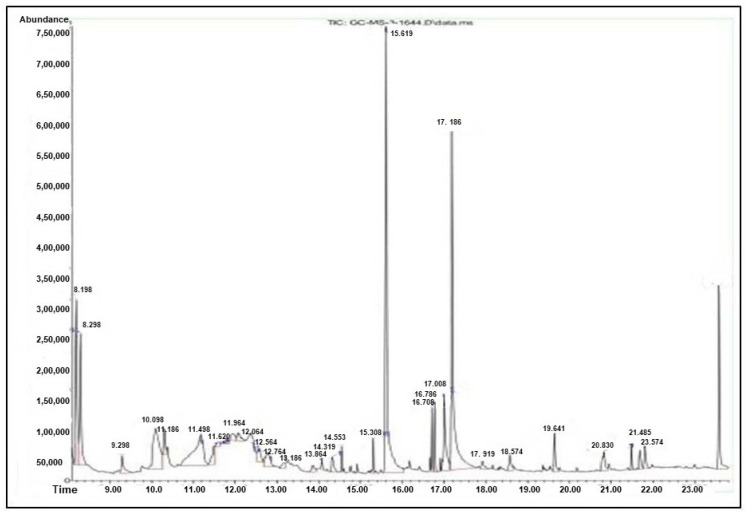
GC–MS analysis of *F. leucopyrus* leaf extract.

**Figure 4:**
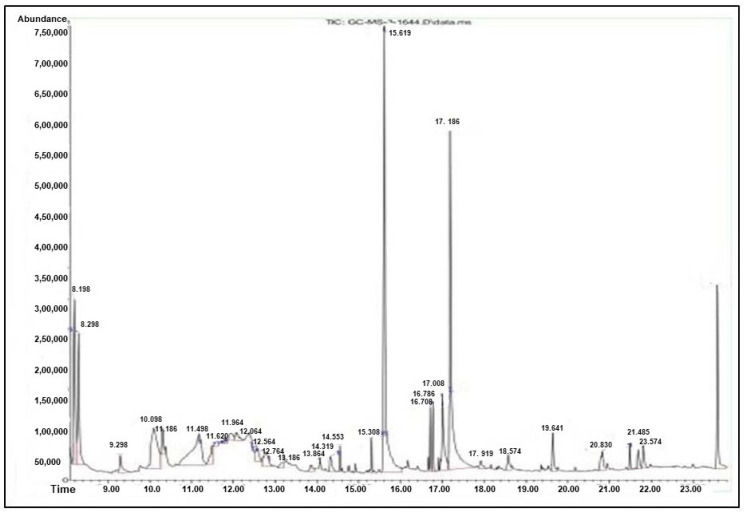
GC–MS result of root extract of *F. leucopyrus*.

Figure 3 and Figure 4 depict chromatograms of the leaf and root extracts of *F. leucopyrus*, which were evaluated by GC–MS. These chromatograms identify the different bioactive and phytoconstituents components present in the extracts. The mass spectrum of compounds detected in the GC–MS mass spectra of extracts of *F. leucopyrus* leaves and roots was compared to the standard compounds spectra provided by the NIST library in Table S2 and Table 3. *F. leucopyrus* aqueous leaf extract contained 30 different bioactive and phytocompounds, as indicated in the GC–MS chromatogram (Figure 3).

The resulting bioactive compounds, such as Furancarboxaldehyde 5-(hydroxymethyl)-,l-[-]-4-Hydroxy-1-methylproline, 2-Methoxy-4-vinylphenol, 1,2,3-Benzenetriol, 1,2,3-Benzenetriol, N-(Cyclohexyl) succinimide, N,N-Dimethyltryptamine, Hexadecanoicacidmethylester, n-Hexadecanoicacid, Phytol, Hexadecanoic acid, 2-hydroxy-1-(hydroxymethyl) ethyl ester, Squalene, and Vitamin E, present in the leaf extract of *F. leucopyrus* were responsible for its antioxidant and anti-inflammatory activities.

Similarly, anti-inflammatory and antioxidant activity was found in the compounds of 2-Furancarboxaldehyde, 5-(hydroxymethyl), 2-Hydroxyphenylacetic acid methylester, N-Ethyl-2- phenethylamine, Dihydrotecomanine, Methyl beta-d-galactopyranoside, beta- (4-Hydroxy-3-methoxyphenyl) propionicacid, n-Hexadecanoicacid, 9,12-Octadecadienoicacid (Z,Z)-, 2H-1-Benzopyran-2-one, 7,8-dihydroxy-6-methoxy, 1H-Indol-4-ol,3-methyl- Pyrazolo[3,4-b] indole-3-carboxylic acid, 1,8-dihydro-,Hexadecanoic acid, 2-hydroxy-1-(hydroxymethyl)ethylester, beta.-Sitosterol, 11-Octadecenoicacid methylester, and Aceticacid3-hydroxy-7-isopropenyl -14a-dimethyl -2,3,4,4a,5,6,7,8-octahydronaphthalen -2-ylesteraqueous root extract *F. leucopyrus*.

### 3.7. In Vitro Antioxidant Activity

We tested the free radical scavenging activity of the *F. leucopyrus* leaf and root aqueous extracts using DPPH radical, superoxide radical, hydroxyl radical, ABTS, and chelating activities, as shown in Table 5 in the form of percentage inhibition (*n* = 3) as well as IC 50 value.

### 3.8. Anti-Inflammatory Activity

Carrageenan-induced edema has been employed frequently in experimental animal models of acute inflammation, and it is thought to be biphasic. Rats (*n* = 6) with carrageenan-induced paw edema had the aqueous extract of the leaves and roots of *F. leucopyrus* tested for anti-inflammatory activity, and it was compared to conventional medications (Indomethacin). Inflammatory reduction capacity in rat paw size increased noticeably after receiving carrageenan subcutaneously, which is a sign of inflammation. Results demonstrated that the injection of carrageenan into the hind paw induced a progressive edema. Also, the aqueous leaf extract of *F. leucopyrus* showed a statistically significant decrease in paw thickness in a dose-dependent manner.

The reference drug indomethacin 3 mg kg^−1^ body weight similarly exhibited a significant dose-dependent decrease in paw edema. At a dose of 500 mg kg^−1^, p.o., the aqueous extract of *F. leucopyrus* root significantly reduced inflammation (*p* < 0.001) when compared to the control. The dose of *F. leucopyrus* root extract that is closest to the standard and has potential anti-inflammatory activity at 2 h is 500 mg kg^−1^ body weight. The outcomes showed that an aqueous extract of the root of *F. leucopyrus* may be beneficial to health, with dose-dependent anti-inflammatory effects. As a result, similar possibilities could be investigated in regard to *F. leucopyrus*. Because free radicals and reactive oxygen species are known to be implicated in the inflammation cascade, the anti-inflammatory effects of the leaf and root extracts were also studied. Aqueous extracts, like conventional indomethacin, showed a dose-dependent reduction in edema, and a direct association between antioxidant and anti-inflammatory activity appeared to exist. The extract’s ability to scavenge reactive oxygen species, which can function as a trigger for an inflammatory response, may be important in decreasing inflammation.

**Figure 5:**
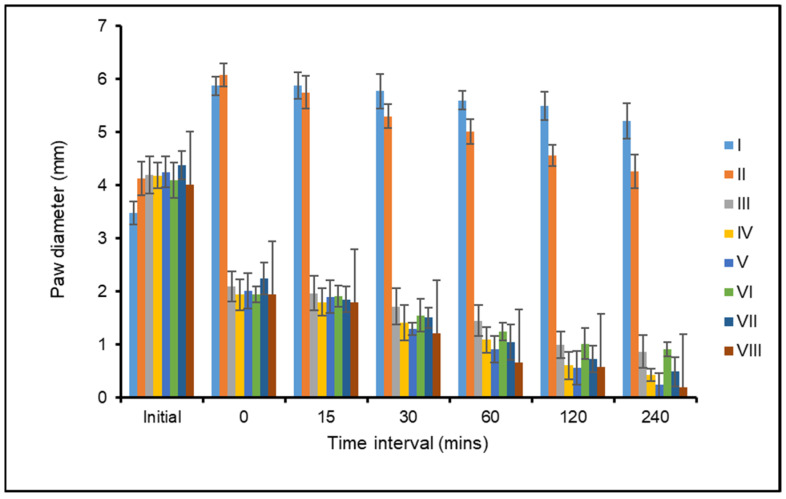
In vivo anti-inflammatory activity of aqueous leaf and root extract of *F. leucopyrus* on carrageenan induced paw edema in rats.

**Figure 6:**
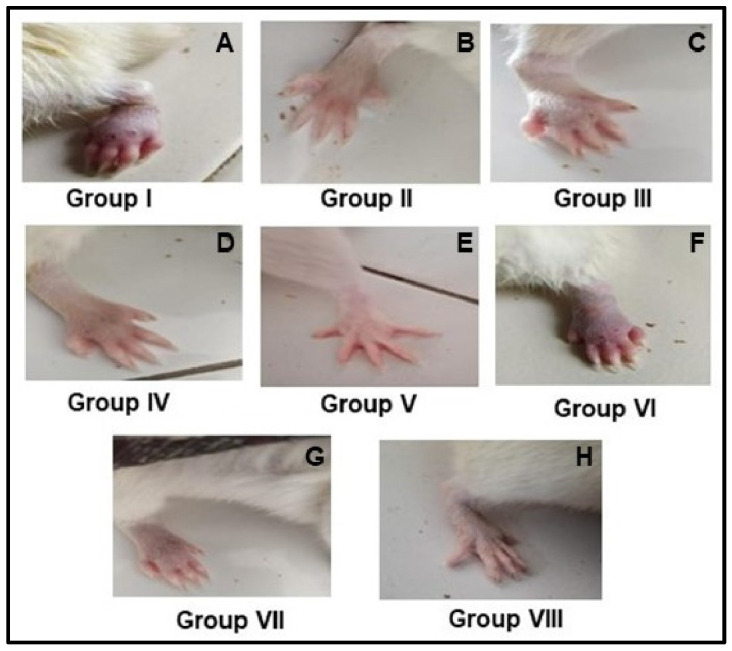
Anti-inflammatory activity of aqueous extract of the leaves and roots of *F. leucopyrus*. The figure illustrates the paw sizes of the rats after receiving treatments. A—the inflammation in the untreated control (Group I); B—shows that inflammation reduced in animals that received the standard drug, Indomethacin (Group II); C, D, E—show that inflammation gradually reduced when increasing the concentration of *F. leucopyrus* leaf extract (Group III—100 mg, Group IV—250 mg, and Group V—500 mg); F, G, H—show that inflammation gradually reduced when increasing the concentration of *F. leucopyrus* root extract (Group III—100 mg, Group IV—250 mg, and Group V—500 mg).

The results were depicted in Figure 5 and Figure 6. In acute inflammation caused by carrageenan, *F. leucopyrus* demonstrated a substantial reduction in paw thickness. Inflammatory rats were given *F. leucopyrus* root extract at doses of 100, 250 and 500 mg kg^−1^ bodyweight, followed by leaf extract, to reduce paw edema.

Note: I—Edema control group, II—edema treated with standard drug (Indomethacin) group, III—edema treated with 100 mg of *F. leucopyrus* leaf group, IV—edema treated with 250 mg of *F. leucopyrus* leaf group, V—edema treated with 500 mg of *F. leucopyrus* leaf group, VI—edema treated with 100 mg of *F. leucopyrus* root group, VII—edema treated with 250 mg of *F. leucopyrus* root group, and VIII—edema treated with 500 mg of *F. leucopyrus* root group. Paw diameter was measured in millimeters, time intervals were checked by minutes.

Maximum inflammation was seen in the control group (untreated) of rats after 15 min, and it remained without further treatment for the next four measurements. This showed an immunological response to acute inflammation as it decreased very gradually across thirty minutes. Compared to the control group, the inflammation was significantly reduced in the group of animals that received an oral administration of aqueous leaf and root extracts of *F. leucopyrus* and standard medicine, indomethacin. Overall, the studied plant extracts significantly and dose-dependently decreased inflammation over time. A dose-dependent impact in reducing total edema was also seen in leaf extract, and it had the second-highest potency, followed by root extract.

## 4. Discussion

To identify adulteration, substitution, and poor treatment, a plant drug’s physico-chemical characteristics should be assessed. Both the water components and the volatile components of the plant are significantly influenced by moisture. The extractive values are helpful for assessing crude pharmaceuticals and learning more about the chemical components of their active compounds that were solvent-extracted from a specific quantity of medicinal plant material. An approximate indicator of the content of a constituent or combination of constituents is the amount of extractive drug yield to a certain solvent [17]. The extractive value and qualitative phytochemical analysis of *F. leucopyrus* leaf and root extracts produced exceptional results when compared to other solvent extracts, making it one of the best solvents for plant extractions due to its low toxicity and inability to cause the extract to complex or dissociate. As a result, the aqueous leaf and root extract of *F. leucopyrus* was employed for additional research, including the discovery of chemical components that are pharmacologically active and a quantitative assessment of the plant. The extraction technique can be used to obtain the bioactive components that are present in the plant and have a significant role in preserving health [18].

One of the pharmacognostic techniques helpful in identifying genuine samples and adulterants is fluorescence analysis. The fluorescence analysis of *F. leucopyrus* aerial parts in powdered form or in various solvents and reagents was assessed in the current investigation [19]. Even though the exact chemicals accountable for the fluorescence properties have been identified in most cases, the procedure’s features of simplicity and speed make it a suitable analytical tool for plant sample identification and raw medicines.

A variety of factors influence the extraction operation’s success, including solvent selection, phytoconstituent chemistry, extraction technique, temperature, and time. The choice of a certain solvent is one of the aspects that is crucial to the extraction process. Differing extraction yields could be the result of the differing properties of the investigated solvents. Since the extraction solvents alter phytochemical composition and yield during the extraction process, it is reasonable to presume that they alter the extract’s pharmacological effect [20]. In the present examination, alkaloids, flavonoids, saponins, and phenols were present in considerable quantity in both the leaf and root extracts. According to the literature, phenolic acids, particularly chlorogenic, caffeic and rosmarinic acids, are the predominant constituents [21].

According to Suresh et al., the stem has the highest percentage (5.78%) of moisture content when compared to leaves and root [22]. The *F. leucopyrus* stem had an organic carbon content of 11.58. The plant’s ethanol extract was discovered to have 2.92 mg kg^−1^ of flavonoids in the root, 2.90 mg kg^−1^ in the stem, and 2.89 mg kg^−1^ in the leaf powder. The plant extract’s phenolic content was discovered to be 3.46 mg kg^−1^. Phenols have stimulating, sanitizing, antiviral, and anti-infectious properties. As per the data from [23] the quantitative analysis, the methanolic extract of the bark and leaves contained 0.02%, 0.19%, and 1.62% of alkaloids, 0.74% saponins, and 1.15% of tannins, respectively.

The compound data of particles inside a specimen can be determined using FT-IR, which uses molecular vibrations to observe molecular bonds in samples absorbing infrared radiation. Since each sample contains different subatomic bonds, FT-IR can be used to determine the types of subatomic bonds present. It is possible to identify the functional groups of active components present in an extract by examining the peak values in the region of IR radiation in the FT-IR spectrum. An FT-IR analysis of the methanol leaf and root extracts of *F. leucopyrus* was conducted in this study and its components were identified based on their absorption peaks. Each component’s spectra revealed significant overlap, indicating a general overlap between some typical absorption peaks of the functional groups in each sample. In this research, the FT-IR analysis of *F. leucopyrus* leaf and root extracts revealed distinct peaks. In the literature, the above characteristics of infrared functional groups were cited as alcohols, alkenes, carbon dioxide, cyclic alkene, nitro compound alkane, aliphatic ether, anhydride, halo compound, aliphatic primary amine, thiol, sulphonyl chloride, and a primary alcohol [24].

The GC–MS analysis of aerial parts of *F. leucopyrus* ethyl acetate extract reported that they include 2-methyl-7-octadecyne, 2-hydroxy-6-methoxy-2H-1-benzopyran-2-one, 2-(9-octadecenyloxy)-ethanol, and (Z)-, dl-alpha. The therapeutic properties of tocopherol, ursodeoxycholic acid, and stigmasterol include antioxidant, anticancer, anti-allergic, and neuro-stimulatory actions, which are consistent with their intended use. Various authors also reported the pharmacological activities of *F. leucopyrus* due to the presence of multiple bioactive compounds. Likewise, 20 chemical compounds were stated in ethanolic leaf extract of *F. leucopyrus* [25]. Ellepola et al. [26] reported that the aqueous extracts of this plant had diuretic activity. According to Soyas et al. [27], this plant has antioxidant and anti-proliferative properties. Unsale et al. [28] investigated this plant’s aphrodisiac effects. Samarakoon et al. [29] demonstrated the cytotoxic and apoptotic effects of a decoction of this plant’s aerial parts.

Natural remedies with anti-inflammatory and antioxidant effects are therefore appealing in this context. Several phytoconstituents have been shown to have antioxidant and anti-inflammatory effects. Based on a number of studies, phytochemicals increase cellular longevity, prevent ageing, and reduce the risk of developing certain inflammatory conditions [30]. This was in line with the research conducted by Gutiérrez et al. [31], which explained that the antioxidant properties of the extract were solely dependent on the alkaloid compounds present in the extract and that phenolic compounds cannot be present in the extract when it is being extracted for its alkaloid content. According to the research, phenolic components and the type of test determine how effective plant extracts are as antioxidants. The antioxidant activity of quercetin, for instance, was extremely low in the FRAP test but extremely high in the DPPH and ABTS assays. In actuality, polyphenols are substances that have several hydroxyl groups joined to one or more benzene rings. Test and phenolic chemicals are typically found as esters or glycosides rather than as free substances. Polyphenols may function as chelating agents, reducing initiators, or by stopping the oxidative processes brought on by singlet oxygen in an active state [32].

Infection has been discovered as a living tissue’s reaction to damage, and it is known to contain a complex array of enzyme activation, mediator release, fluid extravasations, cell migration, tissue disintegration, and repair. The most commonly used primary test to demonstrate novel anti-inflammatory drugs calculates a compound’s capacity to reduce local edema generated in the right paw by injection of an irritating agent. One of the main models used to test new anti-inflammatory medications is carrageenan-induced paw edema, which can result in both local and acute inflammatory reactions. It is a really trustworthy and commonly employed model for paw tissue research during acute inflammation [31]. Cyclooxygenase, which is a recognised target for a number of NSAIDs such as aspirin, plays a substantial role in the conversion of arachidonic acid into prostaglandins in the later inflammatory phase of the carrageenan-induced edema model. Hassan Jamali et al. [33] evaluated the in vivo anti-inflammatory activities of the extract of the seed of *Washingtonia filifera in* rats. Anti-inflammatory activity of the seed extract of *W. filifera* was accessed in carrageenan-induced paw edema and cotton pallet-induced granuloma model in rats (*n* = 6), where it was compared with aspirin.

Higher doses had a slightly lower effect than the standard diclofenac Na drug, which is an anti-inflammatory drug in the class of non-steroidal anti-inflammatory drugs NSAIDS; commonly used as a pain reliever in clinical practice, it inhibits the cyclooxygenase (COX) enzymes, resulting in a reduction in the production of prostaglandins, thromboxanes, and leukotrienes that mediate pain and inflammation [34]. According to research, indomethacin reduces inflammation by preventing the production of prostaglandins by inhibiting the enzyme cyclooxygenase. Additionally, it has been demonstrated that some mediators, including serotonin, bradykinin, and capsaicin, which have been linked to carrageenan-induced paw edema, may be antagonistic to NSAIDs [35].

Our findings were similar to Kumar et al. [36], who reported that the administration of 70% ethanolic extract *of F. leucopyrus* leaf reduces paw edema inflammation in rats at doses of 100, 250 and 500 mg kg^−1^ body weight. The reference medicine indomethacin, at 3 mg kg^−1^ body weight, showed a substantial reduction in paw edema and was dosage dependent. When compared to other dosages and the standard, the 500 mg kg^−1^ body weight of *F. leucopyrus* leaf extract has a potential anti-inflammatory effect at 2 h. The inhibitory activities of protein denaturation, proteinases, and membrane stabilizing activities [37] imply that *F. leucopyrus* extracts have anti-inflammatory potential, which may be related to the phytoconstituents (saponins, phenolics, flavonoids, terpenoids, and alkaloids) contained in the plant. Recent studies have demonstrated the powerful anti-inflammatory potential of these substances [38]. When considered as a whole, it can be said that the aqueous extract of *F. leucopyrus* is the most promising source of anti-inflammatory and antioxidant properties.

## 5. Conclusions

The current study speculates that *F. leucopyrus* aqueous leaf and root extract is a potential source of antioxidants because it showed antioxidative qualities in an antioxidant assay. The extracts from *F. leucopyrus* that were evaluated showed the best anti-inflammatory and antioxidant action because they contained saponins, tannins, flavonoids, and alkaloids. The extract also has anti-inflammatory qualities since it produces effects comparable to those shown in a paw edema model generated by carrageenan. The edema was reduced by the extracts in a dose-dependent manner. The usage of herbal products with anti-inflammatory and antioxidant components could be a viable choice given the growing need for alternatives to control the many kinds of pro-oxidative and inflammatory processes. The traditional usage of this plant for therapeutic purposes was supported by this investigation. These findings enable us to draw the conclusion that *F. leucopyrus* has the potential to be used in the creation of novel anti-inflammatory therapies. To fully understand the precise mechanism of action of the bioactive components of *F. leucopyrus* in inflammatory processes, more research needs be done.

## Figures and Tables

**Table 1:** Physico-chemical characterization of the powdered leaf and root samples of *F. leucopyrus*.

Physico-Chemical Characterization	Values Obtained (%) (*w*/*w*)	
	*Flueggea leucopyrus*	
	Leaf	Root
Moisture content (Loss of drying)	9.12 ± 0.07	13.86 ± 0.12
**Ash values**		
Total ash	3.58 ± 0.13	4.16 ± 0.07
Water-soluble ash	1.26 ± 0.10	2.16 ± 0.21
Acid-insoluble ash	3.61 ± 0.09	3.47 ± 0.08
Sulphate ash	5.44 ± 0.22	5.75 ± 0.14
Fiber content	10.23 ± 0.14	11.63 ± 0.11
**Extractive values**		
Ethanol	9.84 ± 0.12	9.54 ± 0.17
Methanol	12.67 ± 0.08	9.87 ± 0.21
Aqueous	10.63 ± 0.11	14.96 ± 0.15
Petroleum ether	7.34 ± 0.09	5.85 ± 0.10
Chloroform	8.46 ± 0.05	6.25 ± 0.07

Values are means of three independent analyses of the extract ± standard deviation (*n* = 3).

**Table 2:** Fluorescence analysis of leaf and root of *F. leucopyrus*.

Experiments	Leaf			Root		
	Visible Daylight	UV Light		Visible Daylight	UV Light	
		254 nm Short	365 nm Long		254 nm Short	365 nm Long
Powder	Olive green	Light Green	Dark Black	Light Brown	Light Brown	Light Black
Powder + 1 N NaOH	Brown	Dark Green	Black	Dark Brown	Dark Green	Dark Black
Powder + Conc. HCI	Light Green	Dark Green	Dark Black	Dark Brown	Charcoal black	Dark Black
Powder + Conc. H_2_SO_4_	Brown	Green	Black	Light Brown	Dark Green	Black
Powder + 50% HNO_3_	Light Green	Dark Green	Dark Black	Dark Brown	Dark Green	Dark Black
Powder + Acetic acid	Light Green	Light Green	Dark Black	Light Brown	Light Green	Dark Black
Powder + FeCl_3_	Black	Black	Dark Black	Pine Green	Dark Green	Charcoal black
Powder + Petroleum ether	Brown	Light Green	Dark Black	Light Green	Dark Green	Black
Powder + Acetone	Green	Dark Green	Dark Black	Brown	Green	Dark Black
Powder + Chloroform	Dark Brown	Dark Green	Black	Light Brown	Dark Green	Dark Black
Powder + Ethanol	Green	Green	Dark Black	Light Brown	Light Green	Light Black
Powder + Methanol	Dark Green	Dark Green	Dark Black	Light Brown	Light Green	Charcoal black
Powder + Ammonia	Light Green	Light Green	Light Black	Light Brown	Dark Green	Dark Black
Powder + 40%NaOH + 10% Lead acetate	Green	Dark Green	Dark Black	Coffee Brown	Dark Green	Black
Powder + 1N HCl	Dark Green	Dark Green	Black	Dark Green	Dark Green	Light Black

**Table 3:** Quantitative analysis of an aqueous extract of the leaves and roots of *F. leucopyrus*.

Parameter	*F. leucopyrus*	
	Leaf	Root
Alkaloids (mg 100 gm^−1^)	12.78 ± 0.17	18.53 ± 0.12
Flavonoids (mg RE gm^−1^ extract)	45.32 ± 0.16	34.82 ± 0.19
Carbohydrates (mg gm^−1^ extract)	53.62 ± 0.07	71.34 ± 0.09
Protein (mg gm^−1^ extract)	32.67 ± 0.15	25.89 ± 0.12
Total phenolic content (mg/GAE/gm extract)	65.80 ± 0.08	78.90 ± 0.09

RE—Rutin equivalent; GAE—Gallic acid equivalent. Values are means of three independent analyses of the extract ± standard deviation (*n* = 3).

**Table 4:** FT-IR peak values and functional groups of the *F. leucopyrus* leaf and root aqueous extracts.

Bond/Stretching	Frequency (cm^−1^)		Functional Groups
	*F. leucopyrus*		
	Leaf	Root	
O-H stretching	3398.57	3414.00	Alcohols, phenols
H-bonded	-	3784.34, 3730.33	
N-H bend	1624.06	1649.14	Primary amines
H-C=O:C-H stretching	2353.16	2370.51	Aldehydes
C-N stretching	1230.58, 1082.07	1138.00, 1037.70	Aliphatic amines
C-H bend	1454.33	-	Alkanes
	-	2929.87	
C-C stretching	-	1568.13, 1473.62	Aromatics
C-Br stretching	605.65	-	Alkyl halides

**Table 5:** Free radical scavenging activity of aqueous extract of the leaves and roots of *F. leucopyrus*.

Sample Extract Dose (µL mL^−1^)	% of Inhibition				
	DPPH Radical	ABTS	Superoxide Radical	Chelating Activity	Hydroxyl Radical
**Leaf**					
100	43.85 ± 2.749	47.25 ± 1.564	45.25 ± 7.231	42.87 ± 1.433	41.24 ± 5.132
200	55.12 ± 0.987	62.65 ± 2.890	57.35 ± 1.890	53.12 ± 4.901	52.72 ± 2.643
300	63.73 ± 1.890	78.45 ± 6.231	60.49± 1.200	68.53 ± 2.183	68.98 ± 3.764
400	79.22 ± 0.789	81.78 ± 7.679	83.02 ± 3.654	77.87 ± 3.894	89.12 ± 1.539
500	90.45 ± 2.675	86.90 ± 1.432	90.78 ± 2.117	87.30 ± 7.543	91.63 ± 432
IC_50_ (µg mL^−1^)	1.59 ± 0.32	0.82 ± 1.041	1.51 ± 0.98	1.59 ± 0.52	1.63 ± 1.023
**Root**					
100	45.18 ± 0.610	43.65± 5.987	41.35 ± 6.377	46.85 ± 1.840	47.68 ± 7.880
200	55.68 ± 5.574	58.09 ± 7.890	51.46 ± 9.870	56.99 ± 6.189	59.35 ± 1.136
300	64.29 ± 5.694	61.65 ± 4.276	59.89 ± 2.950	62.75 ± 7.340	69.43 ± 1.801
400	72.34 ± 5.640	75.71 ± 2.240	76.15 ± 6.115	78.91 ± 4.896	75.80 ± 6.826
500	81.78 ± 3.207	80.90 ± 1.008	86.45 ± 7.433	84.28 ± 8.024	87.61 ± 5.226
IC_50_ (µg mL^−1^)	1.45 ± 2.011	1.48 ± 2.54	1.86 ± 1.25	1.35 ± 0.256	1.13 ± 1.023

Values are means of three independent analysis of the extract ± standard deviation (*n* = 3).

## Data Availability

The data presented in this study are available in the article and Supplementary Material.

## Supplementary Materials

The following supporting information can be downloaded at https://www.mdpi.com/article/10.3390/antiox13080976/s1: Table S1: Phytochemical analysis of various extracts of leaf and root of *F. leucopyrus*; Table S2: GC–MS analysis of bioactive compounds present in aqueous extract of leaf of *F. leucopyrus*; Table S3: GC–MS analysis of bioactive compounds present in aqueous extract of root of *F. leucopyrus*.
