# Supplementary material for: Chemical Composition Analysis and Assessment of Antioxidant and Anti-Inflammatory Activities of Crude Extract of Flueggea leucopyrus on Carrageenan-Induced Paw Edema in Wistar Albino Rats
Source: Antioxidants (Basel). 2024 Aug 12;13(8):976. doi: 10.3390/antiox13080976 (PMC11351626; doi:10.3390/antiox13080976)
Supplement: Supplementary file 1 [file antioxidants-13-00976-s001.zip › antioxidants-3112993-supplementary.pdf]

Table S1 Phytochemical analysis of various extracts of leaf and root of *F. leucopyrus*

Phytochemicals	Extracts					
	Ethanol		Methanol		Aqueous	
	Leaf	Root	Leaf	Root	Leaf	Root
Alkaloids						
a) Mayers'test	-	-	+	+	+	+
b) Wagner' test	-	-	+	+	+	+
c) Dragendraff's test	-	-	+	+	+	+
Flavonoids	+	+	+	+	+	+
Glycosides	+	+	+	-	-	+
Phenols						
a) Ferric chloride test	+	+	+	-	+	+
b) Lead acetate test	+	-	-	+	+	+
Fixed oils	-	-	-	-	+	+
Phytosterols	-	+	+	+	+	-
Quinones	-	+	-	-	+	+
Saponins	+	+	+	+	+	+
Steroids	+	_	+	+	+	+
Carbohydrates	_	+	+	+	+	+
Tannins	+	_	+	+	+	+
Terpenoids	-	-	+	+	+	-
Xanthoproteins	-	-	-	-	-	-
Starch	+	+	+	-	+	+
Coumarin	-	-	+	+	+	+
Resins	-	-	-	+	+	+
Proteins and free amino acids	+	_	+	+	+	+
Cardiac glycosides	-	-	-	-	+	+
Anthocyanin	-	-	+	+	+	+
Coumarin	-	-	-	-	+	+

Note: + Present; - Absent

Table S2 GC-MS analysis of bioactive compounds present in aqueous extract of leaf of *F. leucopyrus*.

Peak No.	Compound Name	RT (min)	Peak Area %	Molecular Formula	Biological activity	Structure
1.	2-Furancarboxaldehyde, 5-(hydroxymethyl)-	8.198	7.03	$C_6H_6O_3$	*In vitro* antioxidant and antiproliferative activities	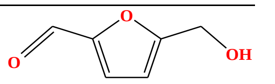
2.	l-[-]-4-Hydroxy-1-methylproline	8.298	6.00	$C_6H_{11}NO_3$	Antioxidant activity	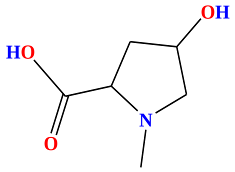
3.	2-Methoxy-4-vinylphenol	9.298	1.24	$C_9H_{10}O_2$	Antioxidant activity	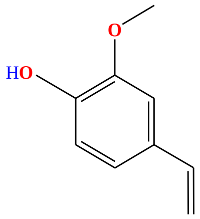
4.	1,2,3-Benzenetriol	10.098	7.25	$C_6H_6O_3$	Antioxidant and antimicrobial activities	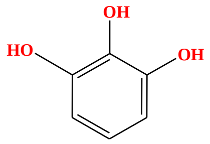
5.	.alpha.-d-Riboside, 1-O-dodecyl-	11.186	7.91	$C_{17}H_{34}O_5$	Antibacterial and antifungal activities	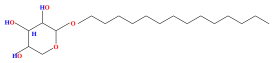
6.	.beta.-D-Ribopyranoside, methyl	11.498	1.43	$C_6H_{12}O_5$	No activity reported	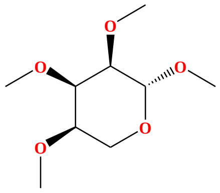
7.	1,2,3-Cyclopentanetriol	11.620	0.88	$C_5H_{10}O_3$	Antimicrobial activity	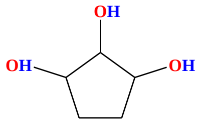
8.	2-Butenoic acid, 2-methyl-, (E)-	11.964	0.83	$C_5H_8O_2$	No activity reported	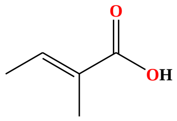
9.	N-(Cyclohexyl)succinimide	12.064	0.65	$C_{10}H_{15}NO_2$	Antioxidant activity	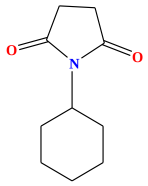

10.	1-Deoxy-d-glucitol	12.564	1.16	$C_6H_{14}O_5$	Antibacterial and antifungal activities	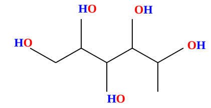
11.	2-Octenoic acid, (E)-	12.764	1.81	$C_8H_{14}O_2$	Antimicrobial and antifungal activities	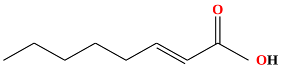
12.	Dodecanoic acid, 1-methylethyl ester	13.186	0.58	$C_{15}H_{30}O_2$	Anti-bacterial activity	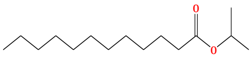
13.	Tetradecanoic acid	13.864	0.51	$C_{14}H_{28}O_2$	Anti-microbial activity	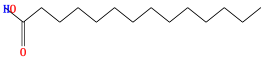
14.	N,N-Dimethyltryptamine	14.319	0.95	$C_{12}H_{16}N_2$	Anti-inflammatory activity	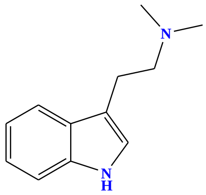
15.	1-Methoxy-3-(2-hydroxyethyl)nonane	14.553	0.61	$C_{12}H_{26}O_2$	Anti-fungal activity	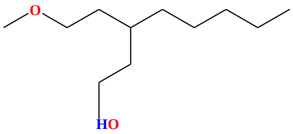
16.	Hexadecanoic acid, methyl ester	15.308	0.91	$C_{17}H_{34}O_2$	Antioxidant and antiproliferative activities	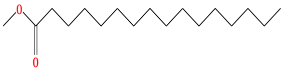
17.	n-Hexadecanoic acid	15.619	19.88	$C_{16}H_{32}O_2$	Anti-inflammatory activity	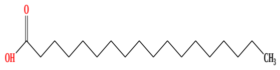
18.	9,12,15-Octadecatrienoic acid, methyl ester, (Z,Z,Z)-	16.708	1.37	$C_{19}H_{32}O_2$	Anti-bacterial activity	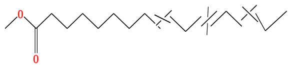
19.	Phytol	16.786	1.70	$C_{20}H_{40}O$	Anti-inflammatory activity	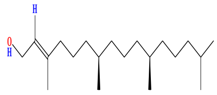
20.	9,12,15-Octadecatrienoic acid, (Z,Z,Z)-	17.008	4.52	$C_{18}H_{30}O_2$	Anti-bacterial activity	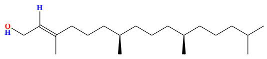
21.	Octadecanoic acid	17.186	17.27	$C_{18}H_{36}O_2$	Anti-bacterial activity	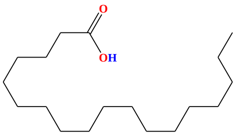
22.	Benzyl.beta.-d-glucoside	17.919	0.57	$C_{13}H_{18}O_6$	Enzymes activities	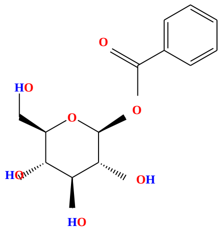
23.	Benzene, 1,1′-(1,2-dimethyl-1,2-ethanediyl)bis-	18.574	0.93	$C_{16}H_{18}$	No Activity	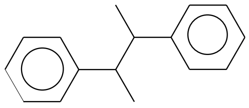

24.	Hexadecanoic acid, 2-hydroxy-1-(hydroxymethyl)ethyl ester	19.641	1.57	$C_{19}H_{38}O_4$	Antioxidant activity	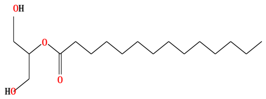
25.	9,12,15-Octadecatrienoic acid, ethyl ester, (Z,Z,Z)-	20.830	1.50	$C_{20}H_{34}O_2$	Antibacterial activity	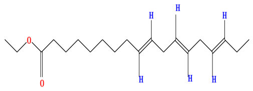
26.	Squalene	21.485	0.65	$C_{30}H_{50}$	Antioxidant and anticancer activities	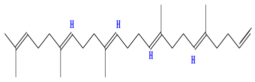
27.	Vitamin E	23.574	7.01	$C_{29}H_{50}O_2$	Antioxidant and anti-inflammatory activities	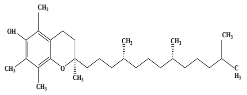

Table S3. GC-MS analysis of bioactive compounds present in aqueous extract of root of *F. leucopyrus*

Peak No.	Compound Name	RT (min)	Peak Area %	Molecular Formula	Biological activity	Structure
1.	2-Furancarboxaldehyde, 5-(hydroxymethyl)-	8.209	6.74	$C_{66}HO_3$	*In vitro* antioxidant and antiproliferative activities	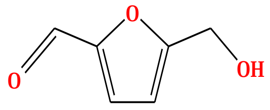
2.	2-Coumaranone	8.353	14.53	$C_8H_6O_2$	Antifungal activity	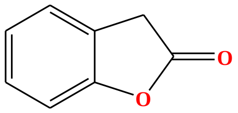
3.	2-Hydroxyphenylacetic acid, methyl ester	9.820	1.09	$C_9H_{10}O_3$	Antioxidant activity	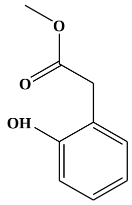
4.	1,2,3-Benzenetriol	10.320	12.25	$C_6H_6O_3$	No activity	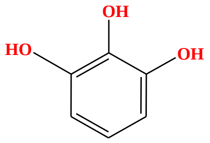
5.	N-Ethyl-2-phenethylamine	11.086	17.54	$C_{10}H_{15}N$	Anti-inflammatory, anti-hypertensive and anti-cancer activities	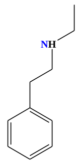
6.	Dihydrotecomanine	11.353	0.76	$C_{11}H_{19}NO$	Anti-inflammatory activity	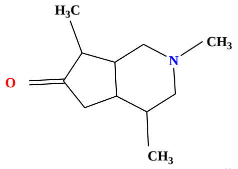
7.	Thiocyanic acid, 2-(2-butoxyethoxy)ethyl ester	11.609	4.38	$C_9H_{17}NO_2S$	Anti-cholesterol and anti-cancer activities	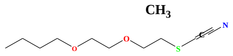
8.	2-Butanamine, N-(1-methylpropyl)-	12.331	1.58	$C_8H_{19}N$	No activity	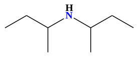
9.	Methyl .beta.-d-galactopyranoside	12.786	9.24	$C_7H_{14}O_6$	Antioxidant and antibacterial activities	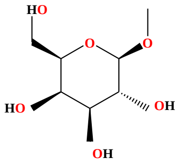
10.	.beta.-(4-Hydroxy-3-methoxyphenyl)propionic acid	13.631	0.81	$C_{10}H_{12}O_4$	Anti-inflammatory and anticancer activities	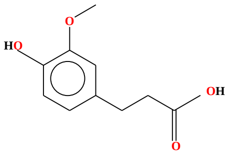

11.	4-((1E)-3-Hydroxy-1-propenyl)-2-methoxyphenol	13.764	1.12	$C_{10}H_{12}O_3$	Antibacterial activity	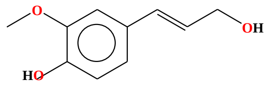
12.	2,4,6-Cycloheptatrien-1-one	15.019	5.11	C_7H_6O	Antibacterial, antifungal and insecticidal activities	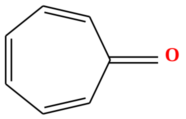
13.	n-Hexadecanoic acid	15.597	3.79	$C_{16}H_{32}O_2$	Anti-inflammatory activity	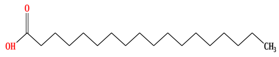
14.	9,12-Octadecadienoic acid (Z,Z)-	16.952	1.09	$C_{18}H_{32}O_2$	Antioxidant, anti-inflammatory and anti-arthritic activities	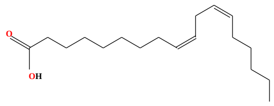
15.	9,12,15-Octadecatrienoic acid, (Z,Z,Z)-	17.008	2.83	$C_{18}H_{30}O_2$	Anti-bacterial activity	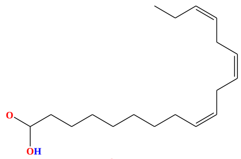
16.	Octadecanoic acid	17.175	2.17	$C_{18}H_{36}O_2$	Antibacterial activity	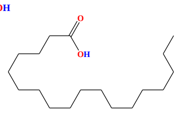
17.	2H-1-Benzopyran-2-one, 7,8-dihydroxy-6-methoxy-	17.241	2.47	$C_{10}H_8O_5$	Anti-inflammatory, anti-arthritic, anti-oxidant and anti-proliferative activities	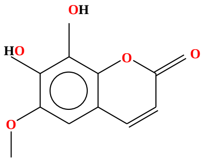
18.	Carbonic acid, monoamide, N-(2-ethylphenyl)-, butyl ester	17.497	0.48	$C_{13}H_{19}NO_2$	No activity	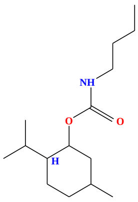
19.	1,3-Cyclohexanedione, 2,2-dimethyl-	18.130	0.68	$C_8H_{12}O_2$	No activity	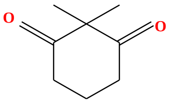
20.	1H-Indol-4-ol, 3-methyl-	18.297	0.71	C_9H_9NO	Anti-cancer, anti-inflammatory, Anti-hypertensive, Anti-tumour and anti-bacterial activities	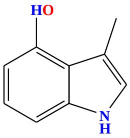

21.	Pyrazolo[3,4-b]indole-3-carboxylic acid, 1,8-dihydro-	18.463	0.76	$C_{10}H_7N_3O_2$	Anticancer, antimicrobial, anticonvulsant, anti-fungal and anti-inflammatory activities	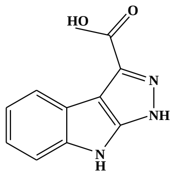
22.	1,1'-Biphenyl, 4,4'-diethoxy-	19.085	1.82	$C_{14}H_{14}O_2$	Anticancer activity	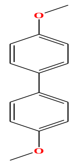
23.	Stigmasterol	19.241	2.26	$C_{29}H_{48}O$	Antimicrobial activity	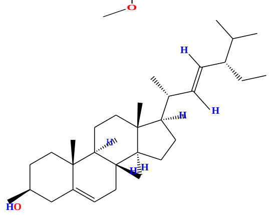
24.	Hexadecanoic acid, 2-hydroxy-1-(hydroxymethyl)ethyl ester	19.630	0.52	$C_{19}H_{38}O_4$	Anticancer and antioxidant activities	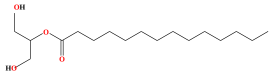
25.	.beta.-Sitosterol	20.541	1.30	$C_{29}H_{50}O$	Anti-inflammatory and anticancer activities	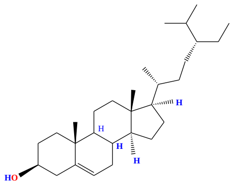
26.	Pentacyclo[9.1.0.0(2,4).0(5,7).0(8,10)]dodecane, 3,3,6,6,9,9,12,12-octamethyl-, anti,syn,anti-	20.630	0.80	$C_{20}H_{32}$	No Activity	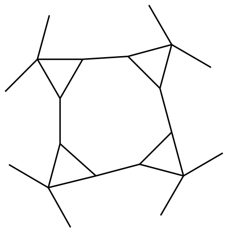
27.	11-Octadecenoic acid, methyl ester	20.785	0.47	$C_{19}H_{36}O_2$	Antimicrobial, antioxidant and anti-inflammatory activities	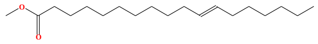
28.	Acetic acid, 3-hydroxy-7-isopropenyl-1,4a-dimethyl-2,3,4,4a,5,6,7,8-octahydronaphthalen-2-yl ester	21.874	1.14	$C_{17}H_{26}O_3$	Anti-inflammatory, antioxidant and antibacterial ac tivities	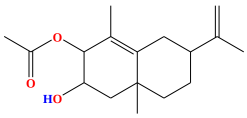
